# Integrated metabolomic and transcriptomic analysis of the anthocyanin regulatory networks in *Salvia miltiorrhiza* Bge. flowers

**DOI:** 10.1186/s12870-020-02553-7

**Published:** 2020-07-23

**Authors:** Tao Jiang, Meidi Zhang, Chunxiu Wen, Xiaoliang Xie, Wei Tian, Saiqun Wen, Ruike Lu, Lingdi Liu

**Affiliations:** 1 Institute of Cash Crops, Hebei Academy of Agricultural and Forestry Sciences, Shijiazhuang, 050051 Hebei China; 2 Institute of Chinese Herbal Medicines, Hubei Academy of Agricultural Sciences, Enshi, 445000 Hubei China

**Keywords:** Medicinal plant, Anthocyanin, Transcriptome, Metabolite, *S. Miltiorrhiza*

## Abstract

**Background:**

The objectives of this study were to reveal the anthocyanin biosynthesis metabolic pathway in white and purple flowers of *Salvia miltiorrhiza* using metabolomics and transcriptomics, to identify different anthocyanin metabolites, and to analyze the differentially expressed genes involved in anthocyanin biosynthesis.

**Results:**

We analyzed the metabolomics and transcriptomics data of *S. miltiorrhiza* flowers. A total of 1994 differentially expressed genes and 84 flavonoid metabolites were identified between the white and purple flowers of *S. miltiorrhiza*. Integrated analysis of transcriptomics and metabolomics showed that cyanidin 3,5-O-diglucoside, malvidin 3,5-diglucoside, and cyanidin 3-O-galactoside were mainly responsible for the purple flower color of *S. miltiorrhiza.* A total of 100 unigenes encoding 10 enzymes were identified as candidate genes involved in anthocyanin biosynthesis in *S. miltiorrhiza* flowers. Low expression of the *ANS* gene decreased the anthocyanin content but enhanced the accumulation of flavonoids in *S. miltiorrhiza* flowers.

**Conclusions:**

Our results provide valuable information on the anthocyanin metabolites and the candidate genes involved in the anthocyanin biosynthesis pathways in *S. miltiorrhiza*.

## Background

*Salvia miltiorrhiza* Burge, known as “Danshen” in China, belongs to the Labiatae family and is a perennial herb from the *Salvia* genus. This plant has been widely cultivated in China for two thousand years. *S. miltiorrhiza* is an important medicinal plant, and the rhizome is used extensively for the treatment of cardiovascular and cerebrovascular diseases [1]. The extract from roots of *S. miltiorrhiza* is composed of many chemical components including phenolic acids, flavonoids, and polysaccharides, which are used in traditional Chinese medicine. In particular, tanshinone, a diterpene quinone extracted from *S. miltiorrhiza*, exhibits a wide range of pharmacological effects and may be useful in the treatment of Alzheimer’s disease [2] and the prevention of osteoporosis [3].

*S. miltiorrhiza* Bge. f. alb is an intraspecific variety of *S. miltiorrhiza* Bge. that is found in Shandong Province in China [4]. Morphological differences in varieties of this species include differences in flower color, e.g., the flower color of *S. miltiorrhiza* Brge. is purple, while that of *S. miltiorrhiza* Bge. *F. alba* is white. Flower color is influenced by many internal and external factors, but anthocyanin content and type are among the most important factors that determine flower color. Anthocyanins that belong to the flavonoid family are important natural colorants widely distributed among leaves, flowers, fruits, and roots. Over 635 anthocyanins have been identified so far [5]. Cyanidin (Cy), delphinidin (Dp), pelargonidin (Pg), peonidin (Pn), petunidin (Pt), and malvidin (Mv) are six common anthocyanin pigments that are the sources of purple, blue, and red colors [6]. The biosynthesis of anthocyanin is catalyzed by a series of enzymes in the phenylpropanoid and flavonoid biosynthetic pathways [7, 8]. The genes involved in the biosynthesis of anthocyanin have been reported in many plant species such as *Arabidopsis* [9], spinach [10], and alfalfa [11]. Studies have found that color mutations are often found in fruits, flowers, and leaves. Color variation can be affected by single-gene mutations in apples [12]. The expression of genes coding for enzymes and transcription factors play multiple key roles in regulating anthocyanin biosynthesis [13]. For example, high expressions of dihydroflavonol 4-reductase (DFR), anthocyanidin synthase (ANS), and anthocyanidin 3-O-glucosyltransferase (UFGT) commonly increase color accumulation in fruits [14], and the R2R3-MYB, basic helix-loop-helix (bHLH), and WD40 transcription factors can form an MBW complex to regulate the biosynthesis of anthocyanin [15–17]. The formation of pigment relies on hydroxylation, glycosylation, methoxylation, and acylation to maintain stability [18, 19]. Some distinctive transporters including GST, the ABC transporter, MATE, and SNARE play important roles in anthocyanin transport in plants [20].

UPLC/ESI-Q TRAP-MS/MS is popular in the field of identification and analysis of plant metabolites and has the advantages of high sensitivity and throughput, fast separation, and wide coverage. So far, this technology has been widely applied to analyze the metabolites in tomato, strawberry, and asparagus [21–23]. In recent years, metabolomics integrated with transcriptomics has been widely used to investigate the biosynthesis of metabolites to reveal the biosynthesis pathways of metabolites in plants [24, 25]. In this study, we conducted research on the regulatory networks of anthocyanin biosynthesis in flowers of *S. miltiorrhiza* using metabolomics and transcriptomics. We aimed to identify different anthocyanin metabolites and to analyze the differentially expressed genes (DEGs) involved in anthocyanin biosynthesis. Our results provide not only candidate genes but also valuable information for metabolic engineering of anthocyanin biosynthesis in flowers of *S. miltiorrhiza*.

## Results

### Measurement of total flavonoids and anthocyanins in flowers of *S. miltiorrhiza*

**Fig. 1:**
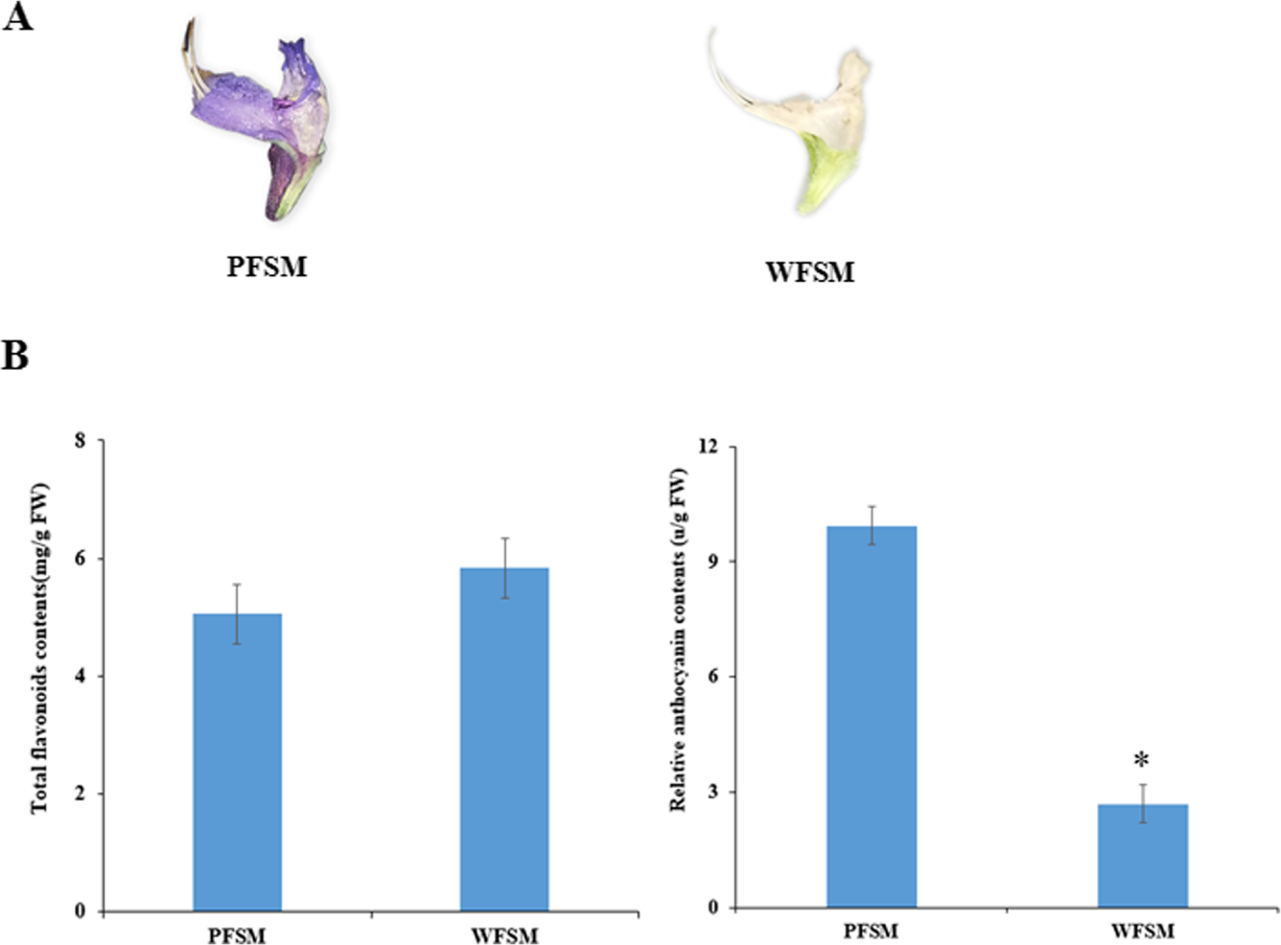
Measurement of total flavonoids and anthocyanins in flowers of *S. miltiorrhiza*

Flavonoids, especially anthocyanins, are the predominant pigment molecules in plants. In our study, the total flavonoids and anthocyanins in *S. miltiorrhiza* flowers were measured. The results showed that the total flavonoid content of WFSM was about 5.83 mg/g of fresh weight, which was higher than the 5.05 mg/g fresh weight of PFSM. The relative anthocyanin content of WFSM was 2.72 units/g of fresh weight, which was significantly lower than the 9.93 units/g fresh weight of PFSM (Fig. 1).

### Flavonoid metabolites in flowers of *S. miltiorrhiza*

**Fig. 2:**
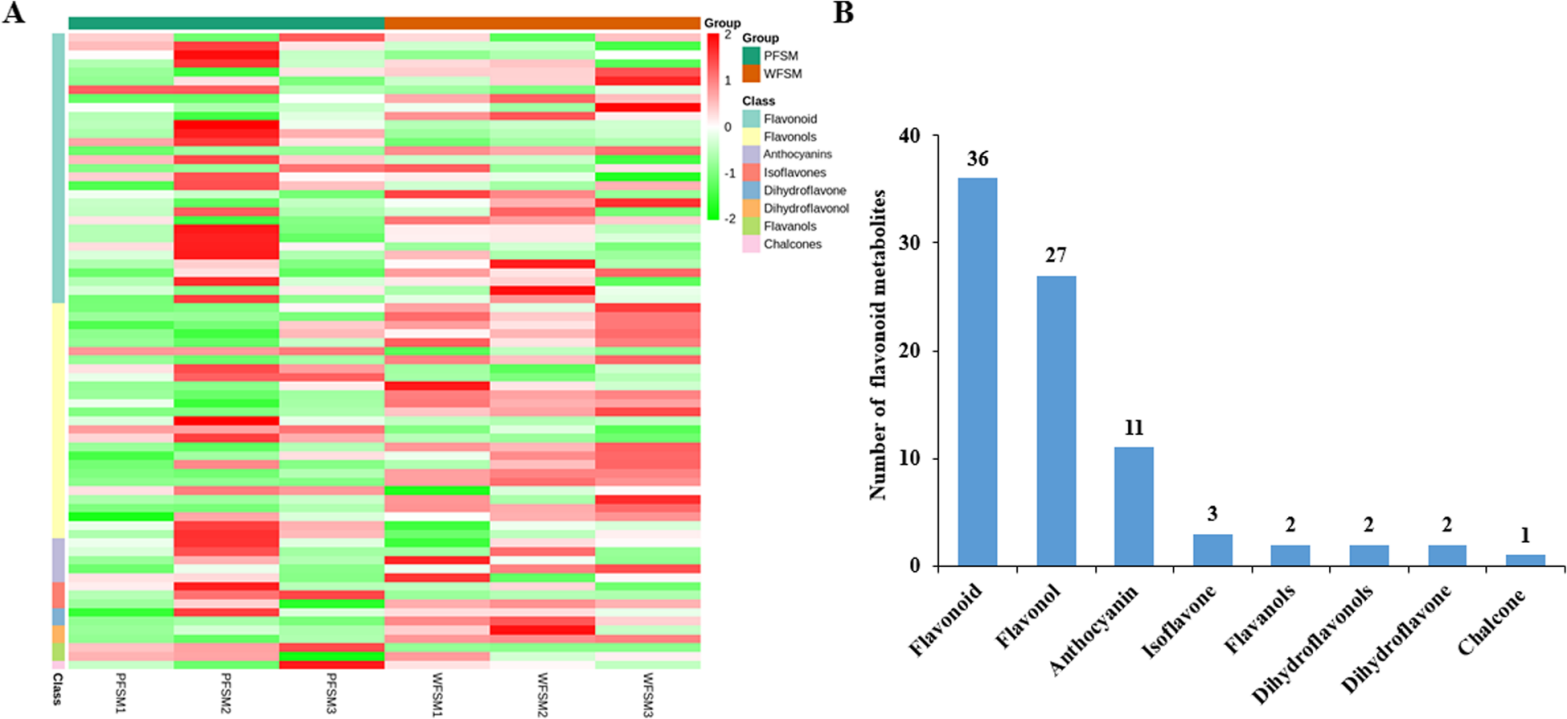
Flavonoid metabolites in PFSM and WFSM

**Fig. 3:**
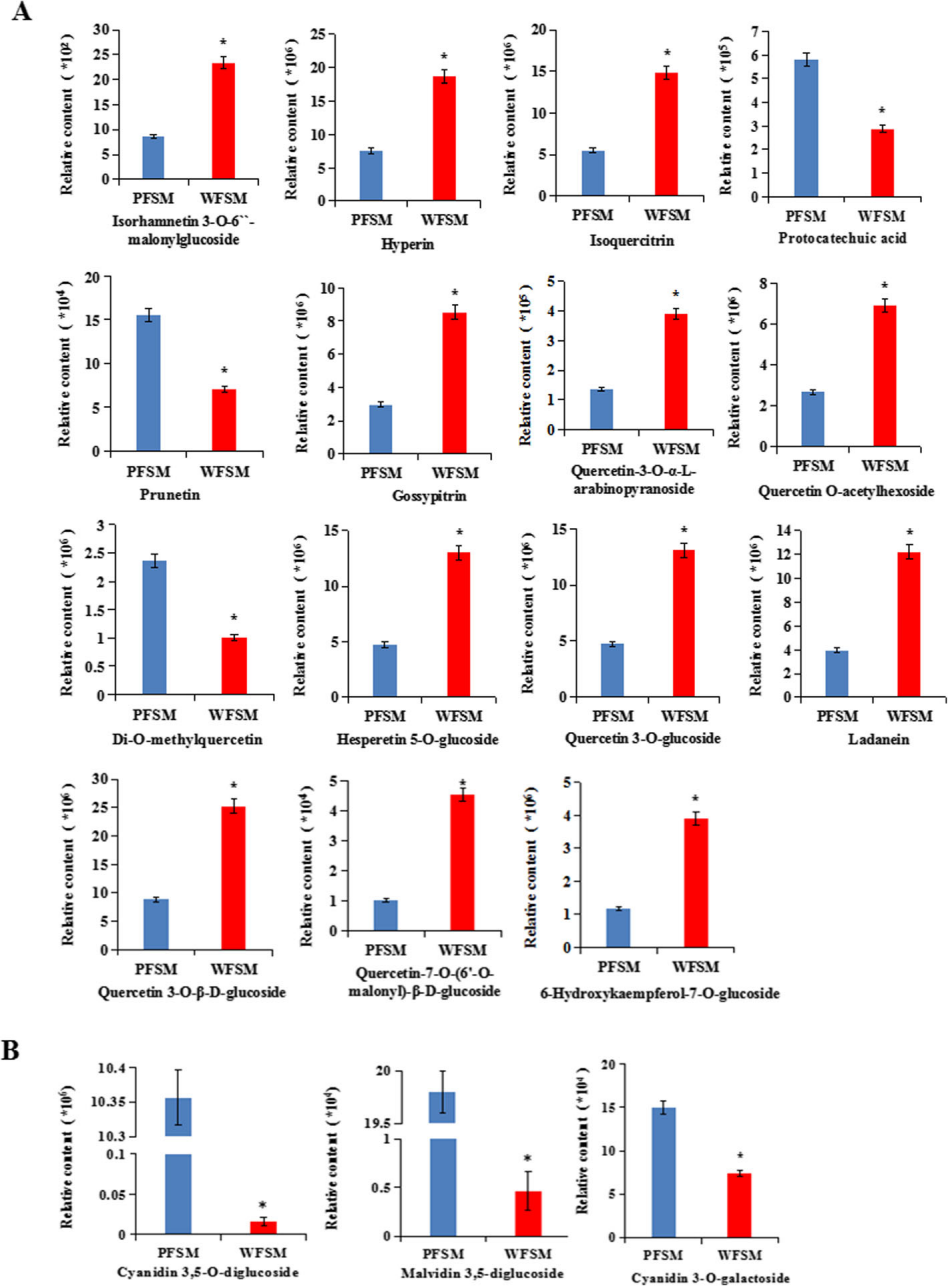
Relative contents of flavonoid and anthocyanin metabolites in PFSM and WFSM

To compare the differential flavonoid metabolites between the white and purple flowers of *S. miltiorrhiza*, the data obtained from UPLC/ESI-Q TRAP-MS/MS were analyzed. In this work, a total of 84 different flavonoid metabolites were identified in flowers of *S. miltiorrhiza* (Table S2). The heatmap of metabolites was drawn by R software after unit variance scaling (UV), and hierarchical cluster analysis (HCA) was performed on the accumulation pattern of metabolites among different samples (Fig. 2a). The 84 flavonoid metabolites were classified into 8 categories, including 36 flavonoids, 27 flavonols, 11 anthocyanins, 3 isoflavones, 2 flavanols, 2 dihydroflavonols, 2 dihydroflavones, and 1 chalcone (Fig. 2b). So far, few studies have qualitatively or quantitatively studied flavonoids from the flowers of *S. miltiorrhiza* or their biosynthesis pathway. From our data, the skeletons of most flavonoids in flowers of *S. miltiorrhiza* included apigenin, quercetin, and kaempferol. Most of the flavonoids in *S. miltiorrhiza* flowers were O-glycosides, with only few being C-glycosides. Among the flavonoid metabolites, isorhamnetin, luteolin, isoquercitrin, quercetin, hyperin, chrysoeriol, apigenin, kaempferol, and their glycosides were abundant in flowers of *S. miltiorrhiza*. Using the identification criterion of the absolute Log_2_FC ≥ 1 and VIP value ≥1, 18 flavonoid contents (21.4% of the total), including 12 upregulated metabolites and 6 downregulated metabolites, were found to be significantly different among the 84 flavonoid metabolites in WFSM vs. PFSM. Among them, quercetin 3-O-β-D-glucoside, hyperin, hesperetin 5-O-glucoside, isoquercitrin, hesperetin 5-O-glucoside, and ladanein demonstrated significantly higher (2.48-fold to 3.11-fold) contents in WFSM vs. PFSM (Fig. 3a).

In total, 11 anthocyanins were identified in flowers of *S. miltiorrhiza*, including cyanidin, petunidin, peonidin, pelargonidin, delphinidin, and malvidin. Among them, the contents of cyanidin 3,5-O-diglucoside, malvidin 3,5-diglucoside, and cyanidin 3-O-galactoside were significantly higher in PFSM vs. WFSM, i.e., 620.16-fold, 42.67-fold, and 2.04-fold, respectively (Fig. 3b). Cyanidin 3,5-O-diglucoside, malvidin 3,5-diglucoside, and cyanidin 3-O-galactoside could be mainly responsible for the purple flower color of *S. miltiorrhiza.*

### Differentially expressed genes in flowers of *S. miltiorrhiza*

**Fig. 4:**
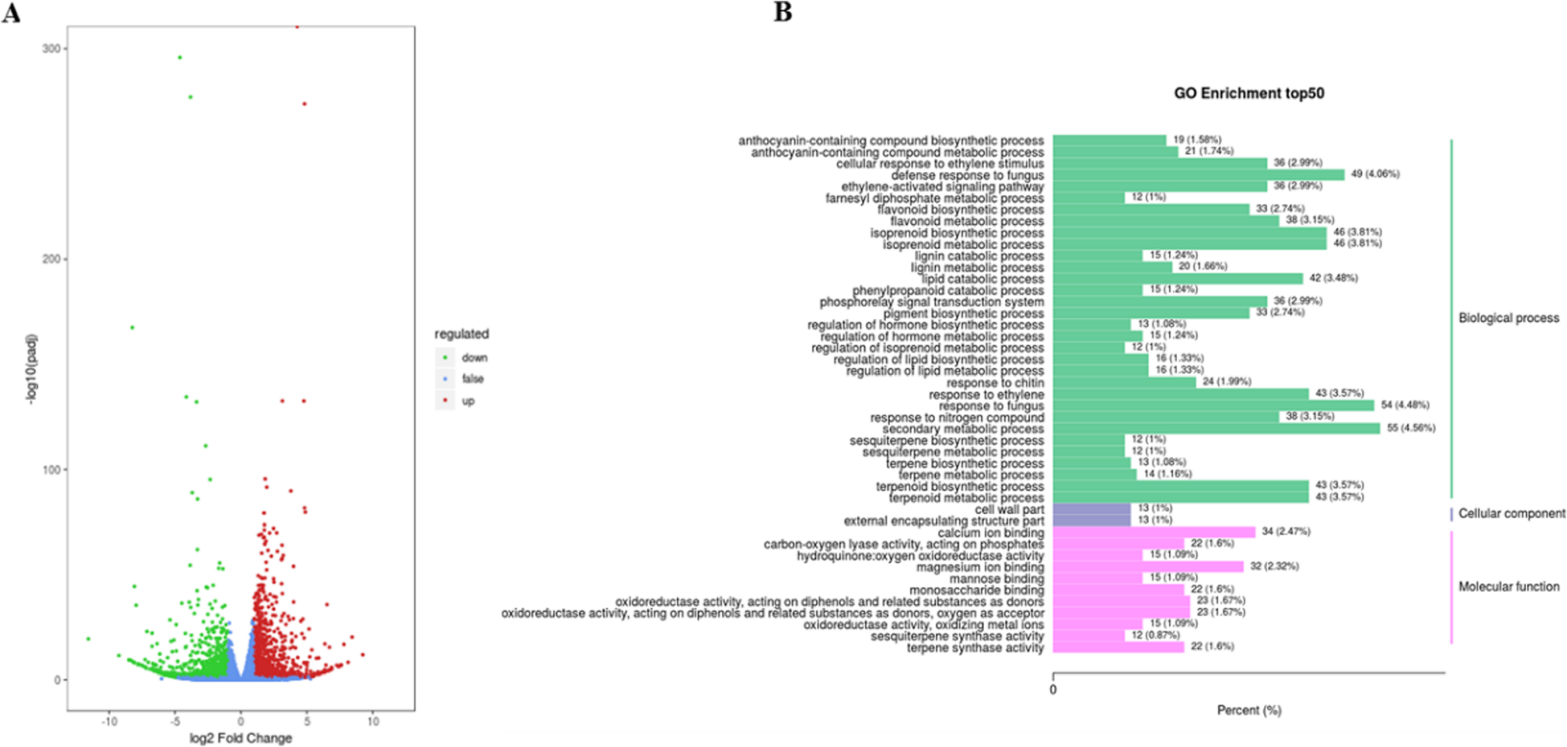
Transcriptome analysis of genes in PFSM and WFSM

To understand the molecular basis of anthocyanin biosynthesis in flowers of *S. miltiorrhiza,* transcriptomes were analyzed to identify differentially expressed genes in the flowers. A total of 48.95 million clean reads were produced from the flowers of *S. miltiorrhiza*. All clean reads were searched in the KEGG, NR, Swiss-Prot, KOG, GO, and Pfam databases with the BLASTX program for functional annotations. A total of 28,539 unigenes were annotated functionally based on the above-mentioned databases. With the filter criteria |Log_2_FC| ≥1 and FDR < 0.05, 1994 differentially expressed genes (DEGs) were identified in the flowers of *S. miltiorrhiza*, including 1173 upregulated genes and 821 downregulated genes in WFSM vs. PFSM (Fig. 4a and Table S3). In GO enrichment analysis of DEGs, 1304 DEGs out of 1994 were involved in three major GO categories, i.e., biological processes, cellular components, and molecular functions, and 19 anthocyanin biosynthetic processes and 33 flavonoid biosynthetic processes were identified in the biological process category (Fig. 4b).

**Fig. 5:**
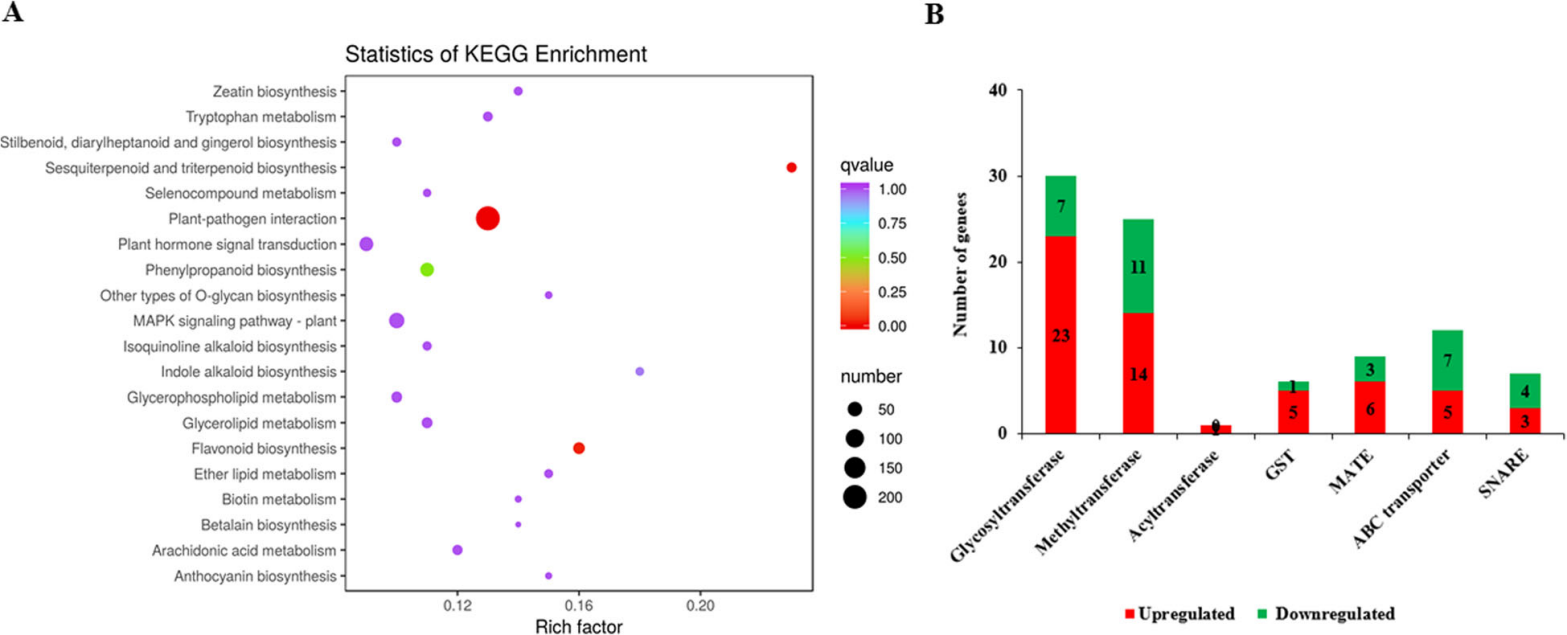
Analysis of genes in PFSM and WFSM

To understand their biological functions and gene interactions, 807 of 1994 DEGs were annotated by the KEGG database. KEGG metabolic pathway enrichment analysis using a Q-value < 0.05 showed the DEGs were enriched in many metabolic processes that included flavonoid and anthocyanin biosynthesis pathways (Fig. 5a). Among these 1994 DEGs, we found 30 glycosyltransferase genes, 25 methyltransferase genes, and 1 acyltransferase gene that catalyzed the synthesis of different types of flavonoids and anthocyanins. In further analysis of the DEGs, we also detected 6 GST genes, 12 ABC transporter genes, 9 MATE genes, and 7 SNARE genes, which might play important roles in transporting anthocyanin to plant vacuoles (Fig. 5b).

### Combined transcriptome and metabolome analysis revealed the biosynthesis of anthocyanin in flowers of *S. miltiorrhiza*

**Fig. 6:**
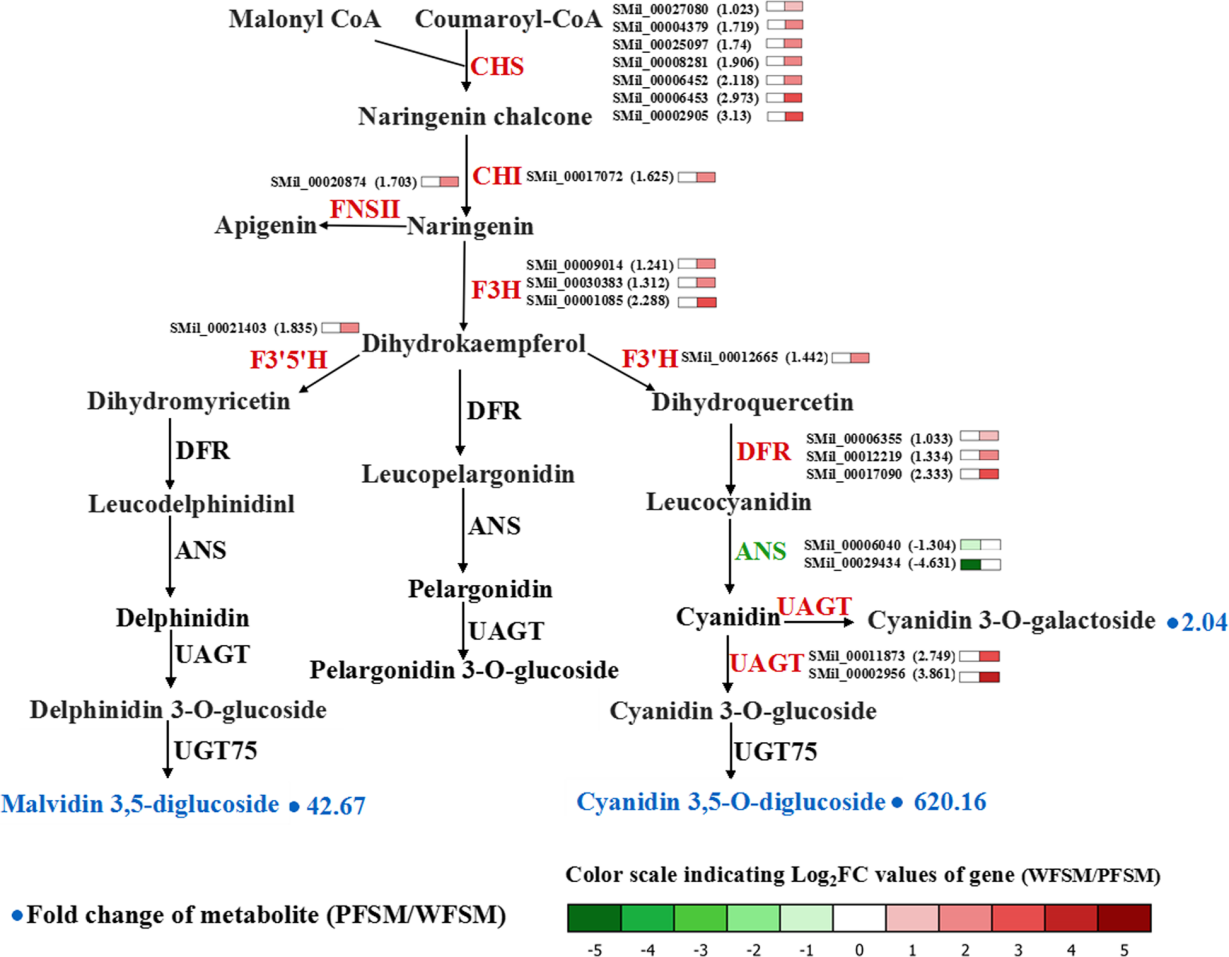
Biosynthetic pathway of anthocyanin in PFSM and WFSM

We combined the analysis of transcriptomic and metabolomic data to understand the pigment biosynthesis pathway in flowers of *S. miltiorrhiza*. The result demonstrated that a large number of flavonoids and anthocyanins were detected in flowers of *S. miltiorrhiza*. Among them, the flavonoid (catalyzed by CHS, CHI, or F3’H) content was high, whereas anthocyanin (catalyzed by ANS) content was lower in WFSM vs. PFSM. To understand this finding, the transcriptome of WFSM was compared with that of PFSM. Analysis of unigenes involved in flavonoids, especially the anthocyanin biosynthesis pathway, was performed to mine the key genes in the metabolism of purple and white flowers of *S. miltiorrhiza*. In total, 100 unigenes that encoded 10 enzymes in the flavonoid and anthocyanin biosynthesis pathways were studied (Table 1). Twenty-one key unigenes had different expression levels; there were 19 upregulated and 2 downregulated unigenes in WFSM vs. PFSM. The core genes in the anthocyanin pathway were analyzed in detail, and the results showed the early genes (*CHS*, *CHI*, etc.) or late genes (*DFR*, *UFGT*, etc.) had higher expression levels except the *ANS* gene in white compared with purple flowers. Among the DEGs, 7 *CHS*, 1 *CHI*, 3 *F3H*, 1 *F3’H*, 1 *F3`5’H*, 1 *FNS* II, 3 *DFR*, and 2 *UAGT* genes were upregulated by 1.033- to 3.13-fold (Log_2_FC), whereas 2 *ANS* genes (SMil_00006040 and SMil_00029434) were downregulated by − 1.304- to − 4.631-fold (Fig. 6 and Table S4). These DEGs affected the biosynthesis of anthocyanin in flowers of *S. miltiorrhiza*.

**Table 1:** Candidate genes related to anthocyanin biosynthesis in flowers of *S. miltiorrhiza*

Gene	Enzyme	No. All^**a**^	No. Up^**b**^	No. Down^**c**^
CHS	Chalcone synthase	9	7	0
CHI	Chalcone isomerase	11	1	0
F3H	Flavonone 3-hydroxylase	32	3	0
F3′H	Flavonoid 3′-monooxygenase	15	1	0
F3’5’H	Flavonoid 3′,5′-hydroxylase	4	1	0
FNS II	Flavone synthase II	1	1	0
DFR	Dihydroflavonol 4-reductase	5	3	0
ANS	Anthocyanidin synthase	7	0	2
UAGT	Anthocyanidin 3-O-glucosyltransferase	15	2	0
UGT75	Anthocyanidin 3-O-glucoside 5-O-glucosyltransferase	1	0	0

No. All^a^, the total number of genes

No. Up^b^, the number of upregulated genes

No. Down^c^, the number of downregulated genes

### The key genes responsible for anthocyanin biosynthesis in flowers of *S. miltiorrhiza*

**Fig. 7:**
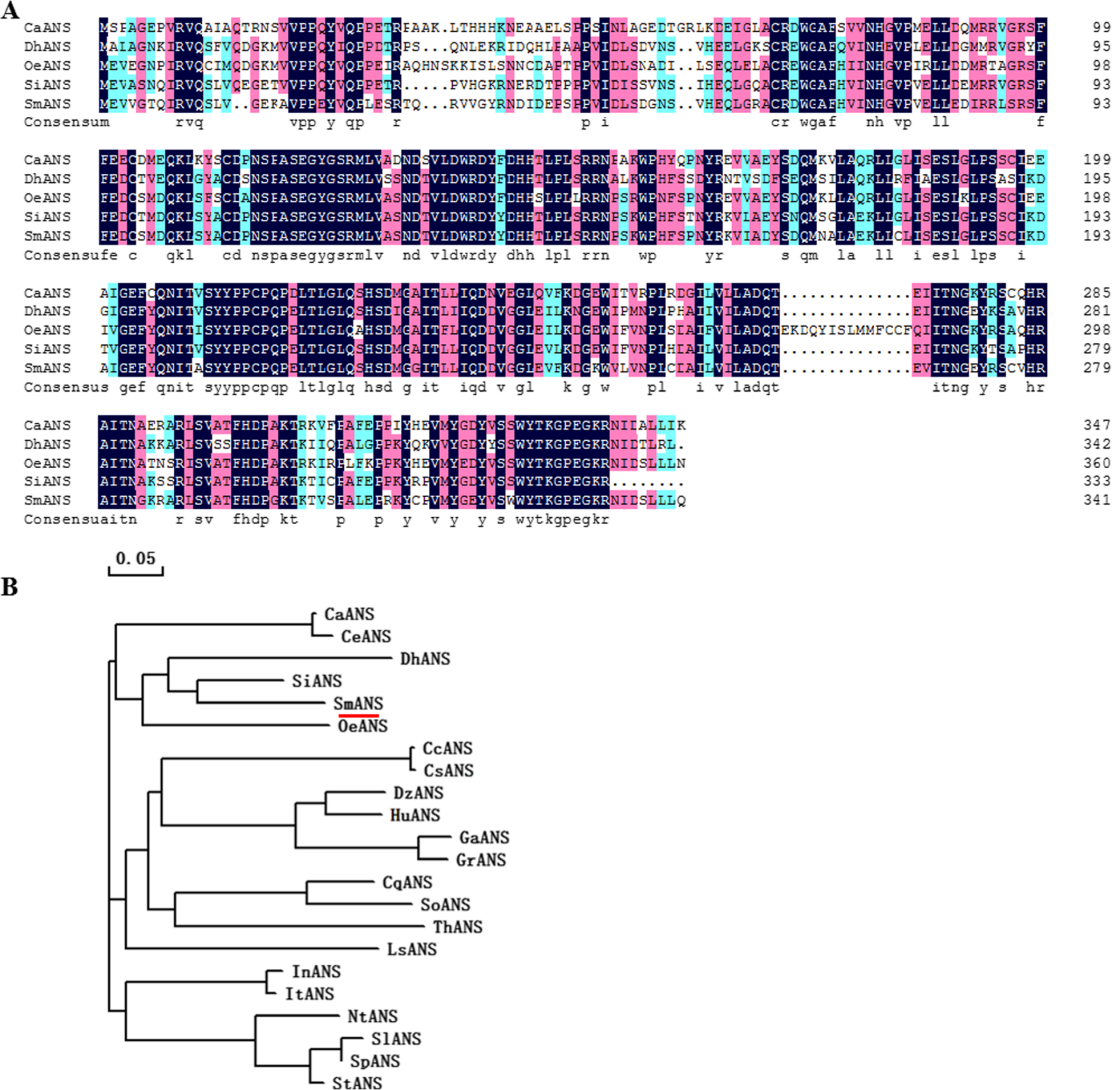
Sequence alignment and phylogenetic tree of SmANS protein (SMil_00006040)

The 2 differentially expressed *ANS* genes were further assigned to the *ANS* family in the NCBI database. Blast analysis indicated that the SMil_00029434 protein had 99.41% sequence identity to SmANS (*S. miltiorrhiza*, AWX67417.1), while the SMil_00006040 protein exhibited 81.19, 71.01, 69.3, and 68.59% amino acid identity to SiANS (*Sesamum indicum*, XP_020551541.1), OeANS (*Olea europaea var. sylvestris*, XP_022887681.1), DhANS (*Dorcoceras hygrometricum*, KZV49046.1), and CaSnRK1 (*Coffea arabica*, XP_027087242.1), respectively (Fig. 7a). Phylogenetic analysis revealed that the SMil_00006040 protein had a close relationship with the ANS protein of *Sesamum indicum* and *Olea europaea var. sylvestris* (Fig. 7b). The sequence of the SMil_00006040 gene was not reported in the NCBI database. We believe the SMil_00006040 gene is a new *ANS* gene in *S. miltiorrhiza*.

### Transcription factors of anthocyanin biosynthesis in flowers of *S. miltiorrhiza*

Transcription factors participate in anthocyanin biosynthesis processes by regulating the gene expression in plants. In our data, 90 transcription factors with different expression levels were related to anthocyanin biosynthesis (Table 2). Among these transcription factors, 14 genes were annotated as MYB-related genes, including MYB36, MYB44, MYB113, and other MYB-dominant proteins. Nine genes encoding bHLH, 1 WD40 gene, and other transcription factors were also found in this study. These transcription factors may contribute to anthocyanin metabolite biosynthesis in the flowers of *S. miltiorrhiza*.

**Table 2:** Transcription factors of anthocyanin biosynthesis in flowers of *S. miltiorrhiza*

Gene	Enzyme	No. All^**a**^ DEGs	No. Up^**b**^ DEGs	No. Down^**c**^ DEGs
MYB	MYB TF	14	10	4
bHLH	Basic helix-loop-helix protein	9	6	3
WD40	WD40 repeat protein	1	0	1
AP2/ERF	Ethylene-responsive TF	33	30	3
WRKY	WRKY DNA-binding protein	4	4	0
MADs	MADS-box TFs	5	4	1
NAC	NAC domain containing protein	4	2	2
Zinc-finger	Zinc finger protein	12	10	2
bZIP	Basic leucine zipper	5	3	2
LBD	Lateral organ boundary domain	3	2	1

No. All^a^ DEGs, the total number of DEGs

No. Up^b^ DEGs, the number of upregulated DEGs

No. Down^c^ DEGs, the number of downregulated DEGs

### QRT-PCR of the transcriptomic data

**Fig. 8:**
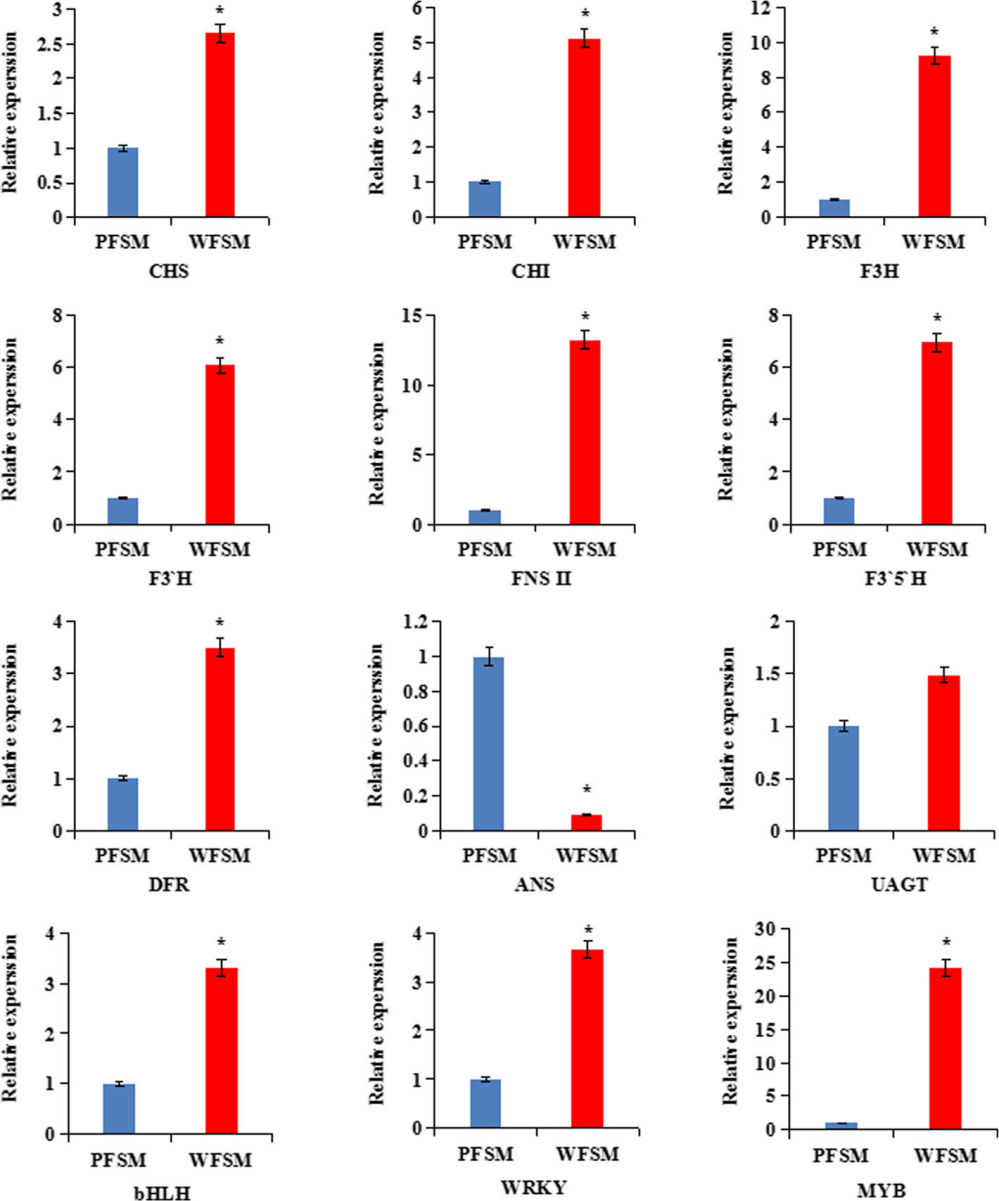
Relative Expression levels of 12 genes involved in the anthocyanin biosynthetic pathway by qRT-PCR analysis

To verify the credibility of the transcriptome information, we further selected 12 DEGs to validate the sequencing results. The qRT-PCR results showed that 11 genes showed higher expression levels and 1 gene had a lower expression in WFSM than in PFSM; our qRT-PCR results were consistent with those obtained with the RNA-Seq method (Fig. 8).

## Discussion

### Anthocyanin identification in the flowers of *S. miltiorrhiza*

Anthocyanins are secondary metabolites in plants such as vegetables, fruits, and medical plants and have many diverse biological functions including antioxidation, regulation of defense responses, and protection against ultraviolet radiation [26]. Anthocyanins are synthesized at the end of the phenylpropanoid metabolic pathway, and the precursors of anthocyanin biosynthesis are malonyl-CoA and coumaroyl-CoA. Most anthocyanins are synthesized through CHS and CHI condensation; F3H, F3’H, or F3`5’H oxidation; or DFR and ANS/LDOX catalysis [27–29]. In our study, in order to elucidate anthocyanin biosynthesis in white and purple flowers of *S. miltiorrhiza*, metabolomic and transcriptomic pathways were analyzed in *S. miltiorrhiza* flowers. A total of 73 flavonoids and 11 anthocyanins were identified in flowers of *S. miltiorrhiza* by UPLC/ESI-Q TRAP-MS/MS (Table S2). We found that the total flavonoid content was higher and the anthocyanin content lower in WFSM vs. PFSM (Figs. 1 and 3). Cyanidin 3,5-O-diglucoside, cyanidin 3-O-galactoside, and malvidin 3,5-diglucoside were significantly different anthocyanin metabolites between WFSM and PFSM. Among the above anthocyanins, the cyanidin 3,5-O-diglucoside content was 620.16-fold higher in PFSM vs. WFSM (Fig. 3b), and the content of cyanidin 3,5-O-diglucoside was also higher than that of other anthocyanins in PFSM. This result suggests that cyanidin 3,5-O-diglucoside is the main anthocyanin in PFSM*.*

Interestingly, we found that cyanidin, petunidin, peonidin, pelargonidin, delphinidin, and malvidin were present not only in PFSM but also in WFSM (Table S2). Quintana et al. [30] suggested that there is a mutation in the early stage of the anthocyanin pathway, leading to no anthocyanidins in white flowers of *Anagallis monellin*. Duan et al. [11] reported that malvidin and petunidin were detected in purple alfalfa flowers, whereas no color anthocyanidins occurred in cream alfalfa flowers. In this study, we found that 6 types of common anthocyanin pigments were present simultaneously in white flowers of *S. miltiorrhiza.* It is possible that the determinants of flower color are involved in the number of pigments, metal ions, or the different molecular conformations of anthocyanin. These pigments are present in small quantities in white flowers of *S. miltiorrhiza*, suggesting that complete anthocyanin metabolic pathways induce anthocyanin biosynthesis in the white flowers of this species*.*

### The genes involved in anthocyanin biosynthesis in flowers of *S. miltiorrhiza*

Transcriptome analysis of the flowers of *S. miltiorrhiza* identified unigenes involved in the anthocyanin biosynthesis process and showed differentially expressed genes. In the anthocyanin biosynthesis pathway, the expression levels of genes encoding CHS, CHI, FNS II, F3H, F3’H, F3`5’H, DFR, and UAGT were higher in WFSM than in PFSM (Fig. 6), which resulted in a higher flavonoid content in the white flowers of *S. miltiorrhiza*. ANS is a key enzyme of the anthocyanin pathway that plays an important role in the conversion of leucoanthocyanidins to anthocyanidins [31]. In this study, we detected two differentially expressed *ANS* genes (SMil_00006040 and SMil_00029434) that were downregulated in WFSM vs. PFSM (Table S4). Blast analysis indicated that the SMil_00006040 gene is a new *ANS* gene in *S. miltiorrhiza*. The low expression level of the *ANS* gene may have inhibited the formation of anthocyanin, leading to the formation of white flowers. Based on the metabolomic and transcriptomic data, we speculated that the low expression of the *ANS* gene decreased the anthocyanin content but enhanced the accumulation of flavonoids in flowers of *S. miltiorrhiza*.

### The function of the ANS gene in flowers of *S. miltiorrhiza*

ANS is one of the four dioxygenases in the anthocyanin biosynthetic pathway that catalyzes the formation of anthocyanidins. Some studies have shown that the absence of *ANS* and *DFR* genes in the anthocyanin biosynthesis pathway would lead to the loss of pigmentation [32, 33]. The white-fruited *Duchesnea indica* (Rosaceae) phenotype is related to the downregulation of the *ANS* gene [34]. The suppressed expression of the *ANS* gene leads to a lack of anthocyanins in Caryophyllales [35]. Li et al. [36] reported that overexpression of *SmANS* increased anthocyanin content but reduced the biosynthesis of salvianolic acid B in *S. miltiorrhiza.* White-flowered *S. miltiorrhiza* is a variety of the *S. miltiorrhiza* species. Our analysis suggested that the white-flowered character was due to the low expression of the *ANS* gene. Next in a future study, we will overexpress *ANS* genes (SMil_00006040 and SMil_00029434) to research their functions in white-flowered *S. miltiorrhiza* (Fig. 7).

### Transcription factors related to anthocyanin biosynthesis

Anthocyanin biosynthesis is mostly regulated by transcription factors at the transcription level. To date, transcription factors of Myb, bHLH, WD40, zinc finger, MADs, and WRKY proteins have been found to regulate anthocyanin biosynthesis [37, 38]. Among them, MYB transcription factors play a key role in the regulation of anthocyanin biosynthesis. In particular, MYB75/PAP1 is a master regulator controlling anthocyanin biosynthesis in *Arabidopsis* [39]. In the anthocyanin biosynthetic pathway, anthocyanin biosynthesis is controlled by the MYB-bHLH-WD40 (MBW) complex in plant species such as *Arabidopsis* [26], maize [40], and cauliflower [41]. In addition to MYBs, the zinc-finger transcription factor (AtZAT6) is also involved in anthocyanin synthesis through direct binding to the promoters of several genes in *Arabidopsis* [42]. The bHLH transcription factors have been proved to positively regulate the biosynthesis of anthocyanin in *Arabidopsis* [43]. MdWRKY11 can increase the expression of F3H, FLS, DFR, ANS, and UFGT to promote anthocyanin accumulation in apples [44]. The *IbMADS10* gene regulates anthocyanin biosynthesis to increase the accumulation of anthocyanin pigments in sweet potato [45]. In our study, we analyzed the transcriptome data and found that 90 important transcription factors including MYB, AP2/ERFs, WRKY, bHLH, MADs, WD40, zinc-finger, NACs, bZIP, and LBD showed significantly different expression levels between WFSM and PFSM (Table 2). We speculated that these differentially expressed transcription factors may be candidate regulators of anthocyanin biosynthesis in *S. miltiorrhiza* flowers.

## Conclusions

We used metabolomics and transcriptomics to reveal the anthocyanin biosynthesis metabolic pathway. A total of 1994 differentially expressed genes and 84 different flavonoid metabolites were identified in two varieties of *S. miltiorrhiza*. In an analysis of the anthocyanin biosynthesis pathway, we identified 100 unigenes encoding 10 enzymes that are involved in anthocyanin biosynthesis in flowers of *S. miltiorrhiza.* Integrated analysis of transcriptomic and metabolomic data showed that the low expression of the *ANS* gene decreased the anthocyanin content but enhanced the accumulation of flavonoids in white flowers of *S. miltiorrhiza*. Cyanidin 3,5-O-diglucoside, malvidin 3,5-diglucoside, and cyanidin 3-O-galactoside are mainly responsible for the purple flower color of *S. miltiorrhiza.* Our results provide valuable information on the anthocyanin metabolites and the candidate genes involved in the anthocyanin biosynthesis pathways in *S. miltiorrhiza*.

## Methods

### Plant materials

White-flowered *S. miltiorrhiza* (WFSM) and purple-flowered *S. miltiorrhiza* (PFSM) were grown at the *S. miltiorrhiza* germplasm resource center at the Institute of Cash Crops, Hebei Academy of Agricultural and Forestry Sciences, China. Fresh flowers were collected from healthy plants in August 2019. All materials were frozen in liquid nitrogen and stored at − 80 °C for RNA and metabolite extraction. All experiments were performed in three biological replicates in this study.

### Measurement of total flavonoid content

Approximately 2.5 g of *S. miltiorrhiza* flowers was used to measure the total flavonoid content by the aluminum nitrate colorimetric method. In brief, 0.5 mL of crude flower extract was mixed with 5.5 mL of 50% ethanol and l mL of 5% NaNO_2_ solution. Then, 1 mL of 10% A1(N0_3_)_3_ solution was added after 6 min of incubation, and the mixture was incubated for another 6 min. Subsequently, 10 mL of 4% NaOH solution and 7 mL of 50% ethanol were added. The final volume of the mixture was 25 mL. The mixture stood for 15 min, and then the absorbance was measured at a wavelength of 506 nm by an ultraviolet spectrophotometer (V-5100B, METASH, Shanghai, China). Rutin was used as a standard solution to prepare a calibration curve, and the results were expressed as rutin equivalent on a fresh weight basis [46].

### Measurement of relative anthocyanin content

*S. miltiorrhiza* flowers (0.1 g) were ground with 1 mL of methanol (0.1% HCl) and were washed twice into 10 mL centrifuge tubes. The final volume of the samples was 5 mL (including methanol (0.1% HCl)). The tissue homogenates were oscillated for 30 s and centrifuged at 4 °C and 12,000 g for 10 min, and the absorbance of the supernatants was measured at a wavelength of 530 nm using an ultraviolet spectrophotometer (V-5100B, METASH). The relative anthocyanin concentration was calculated using the following formula: Q = V * A530 / M (units/g FW), where V represents the volume of the solution, and M represents the weight of the sample. Methanol (0.1% HCl) was used as a blank control [47].

### Metabolite extraction

Freeze-dried flowers were crushed using a mixer mill (MM 400, Verder Retsch, Shanghai, China) with a zirconia bead for 1.5 min at a frequency of 30 Hz. Then, 100 mg powder was weighed and extracted overnight at 4 °C with 1.0 mL 70% methanol aqueous solution (V/V = 70%). Following centrifugation at 10,000 g for 10 min, the extracts were absorbed by a CNWBOND Carbon-GCB SPE cartridge (250 mg, 3 mL; ANPEL, Shanghai, China, www.anpel.com.cn/cnw) and filtered through a 0.22-μm microfiltration membrane (SCAA-104; ANPEL, Shanghai, China, http://www.anpel.com.cn/) before UPLC-MS/MS analysis.

### Ultra-performance liquid chromatography (UPLC) conditions

A UPLC-ESI-MS/MS system (UPLC, Shim-pack UFLC SHIMADZU CBM30A system, Shanghai, China, www.shimadzu.com.cn/) was used to analyze the sample extracts. The UPLC analysis was performed under the following conditions, UPLC: column, Waters (Shanghai, China) ACQUITY UPLC HSS T3 C18 (1.8 μm, 2.1 mm*100 mm); solvent system, water (0.04% acetic acid): acetonitrile (0.04% acetic acid); gradient program, 95:5 V/V at 0 min, 5:95 V/V at 11.0 min, 5:95 V/V at 12.0 min, 95:5 V/V at 12.1 min, 95:5 V/V at 15.0 min; flow rate, 0.40 mL/min; temperature, 40 °C; injection volume: 2 μL. The effluent was alternatively connected to an ESI-triple quadrupole-linear ion trap (Q TRAP)-MS.

### ESI-q trap-MS/MS

Linear ion hydrazine-flight time (LIT) and triple quadrupole (QQQ) scans were conducted on a triple Q TRAP, API 6500 Q TRAP LC/MS/MS system (Applied Biosystems, Shanghai, China) equipped with an ESI turbo ion-spray interface, operating in positive ion mode and negative ion mode. The system was controlled by Analyst 1.6 software (AB Sciex, Shanghai, China). The ESI source was set with the following parameters: ion source, turbo spray; source temperature 500 °C; ion spray voltage (IS) 5500 V. The ion source gas I (GSI), gas II (GSII), and curtain gas (CUR) were set at 55.0 psi, 60.0 psi, and 25.0 psi, respectively; the collision gas (CAD) was high. Instrument tuning and mass calibration were performed with 10 and 100 μmol/L polypropylene glycol solutions in QQQ and LIT modes, respectively. QQQ scans were acquired as multiple reaction monitoring (MRM) experiments with collision gas (nitrogen) set to 5 psi. Declustering potential (DP) collision energy (CE) measurements for individual MRM transitions were completed with further DP and CE optimization. A specific set of MRM transitions was monitored for each period according to the metabolites eluted within the period.

### Identification and quantitative analysis of metabolites

Base on the stepwise MIM–EPI (multiple ion monitoring-enhanced product ions) to build the commercially available standard Metabolites Database (Metware Biotechnology Co., Ltd. Wuhan, China) and the public metabolite databases such as MassBank, KNAPSAcK, HMDB, MoToDB, and METLIN, qualitative analysis of the metabolite data was performed. The quantitative analysis of metabolites used multiple reaction monitoring [48, 49]. Unsupervised PCA (principal component analysis), HCA (hierarchical cluster analysis), and OPLS-DA (partial least-squares discriminant analysis) were performed by the statistics function prcomp within R (www.r-project.org). Significantly different metabolites between groups were determined by VIP ≥1 and an absolute Log_2_FC (fold change) ≥1.

### RNA extraction and Illumina sequencing

Total RNA was extracted from frozen flowers using the RNAprep Pure Plant Kit (Tiangen Biotech, Beijing, China). RNA degradation and contamination were monitored on 1.2% agarose gels. The purified RNA concentrations were quantified using a NanoDropTM 2000 spectrophotometer (Thermo Scientific, Shanghai, China). The quality of the total RNA was examined using an Agilent 2100 Bioanalyzer (Agilent Technologies, Santa Clara, CA, USA). Poly (A) mRNA was enriched from the total RNA using Oligo (dT) magnetic beads. Poly (A) mRNA was subsequently fragmented by an RNA fragmentation kit (Ambion, Austin, TX, USA). The fragmented RNA was transcribed into first-strand cDNA using reverse transcriptase and random hexamer primers. Second-strand cDNA was synthesized using DNA polymerase I and RNase H (Invitrogen, Carlsbad, CA, USA). After end repair and the addition of a poly (A) tail, suitable length fragments were isolated and connected to the sequencing adaptors. The fragments were sequenced on an Illumina HiSeq™ 2500 platform.

### RNA sequencing (RNA-seq) data analysis and annotation

To acquire high-quality reads, the raw reads in fastq format were processed through in-house Perl scripts. Clean reads were obtained from raw data by removing adaptor sequences, low-quality reads, and reads containing ploy-N. All downstream analyses were based on clean, high-quality data. Gene function was annotated using the following: the Kyoto Encyclopedia of Gene and Genome (KEGG) pathway database, the NCBI non-redundant (Nr) database, the Swiss-Prot protein database, the euKaryotic Clusters of Orthologous Groups (KOG) database, the Gene Ontology (GO) database, and the Pfam database.

The levels of gene expression were estimated by RSEM (version 1.2.26) [50]. Analysis of the differentially expressed genes of the two groups was performed with the DESeq R package (1.10.1). DESeq provides statistical routines for determining differentially expressed genes using a model based on the negative binomial distribution. The results of all statistical tests were corrected by multiple tests using the Benjamini and Hochberg false discovery rate. Genes were determined to be significantly differentially expressed at an adjusted *P*-value of < 0.05 according to DESeq. GO enrichment analysis of the differentially expressed genes was implemented by the topGO R package based on the Kolmogorov-Smirnov test. Pathway analysis elucidated significant pathways of differentially expressed genes according to the KEGG database (http://www.genome.jp/kegg/) [51]. We tested the statistical enrichment of differentially expressed genes in KEGG pathways using KOBAS software [52].

### QRT-PCR expression analysis of genes involved in anthocyanin biosynthesis

Total RNA of *S. miltiorrhiza* flowers was reverse-transcribed according to the Quantscript Reverse Transcriptase Kit (Tiangen Biotech, Beijing, China). cDNA was used as a template to measure gene expression. The specific primers for genes involved in anthocyanin biosynthesis and the *S. miltiorrhiza* actin gene (internal control) are listed in Table S1. A quantitative real-time polymerase chain reaction (qRT-PCR) was conducted by a real-time PCR ABI Prism 7500 system (software for 7500 and 7500 Fast Real-Time PCR Systems, V2.0.1, Foster City, CA, USA) using SYBR® Premix Ex Taq™ II (TaKaRa Code No. RR820A, http://www.takarabiomed.com.cn). The comparative CT method (2-^ΔΔCT^ method) was used to quantify gene expression [53].

### Statistical analysis

Statistical analysis was performed using Excel 2010 software (Microsoft Office, USA). Data are presented as means ± standard deviations (SD). The levels of statistical significance were analyzed by the least significant difference (*p* < 0.05).

## Supplementary information

**Additional file 1 Supplemental Table S1**. Sequences of specific primers for qRT-PCR.

**Additional file 2 Supplemental Table S2**. Different flavonoid metabolites in WPSM vs. PFSM.

**Additional file 3 Supplemental Table S3.** Differentially expressed genes in WFSM vs. PFSM.

**Additional file 4 Supplemental Table S4.** Differentially expressed genes of anthocyanin biosynthesis in WPSM vs. PFSM.

## Data Availability

All relevant supporting data sets are included in the article and its supplemental files.

## Abbreviations

Cy: Cyanidin
Dp: Delphinidin
Pg: Pelargonidin
Pn: Peonidin
Pt: Petunidin
Mv: Malvidin
DFR: Dihydroflavonol 4-reductase
ANS: Anthocyanidin synthase
UFGT: Anthocyanidin 3-O-glucosyltransferase
CHS: Chalcone synthase
CHI: Chalcone isomerase
F3H: Flavonone 3-hydroxylase
F3′H: Flavonoid 3′-monooxygenase
F3’5’H: Flavonoid 3′,5′-hydroxylase
FNS II: Flavone synthase II
UGT75: Anthocyanidin 3-O-glucoside 5-O-glucosyltransferase
bHLH: Basic helix-loop-helix
DEGs: Differentially expressed genes
UPLC: Ultra-performance liquid chromatography
LIT: Linear ion hydrazine-flight time
QQQ: Triple quadrupole
GSI: Gas I
GSII: Gas II
CUR: Curtain gas
CAD: Collision gas
MRM: Multiple reaction monitoring
DP: Declustering potential
CE: Collision energy
MIM–EPI: Multiple ion monitoring-enhanced product ions
PCA: Principal component analysis
HCA: Hierarchical cluster analysis
OPLS-DA: Partial least-squares discriminant analysis
KEGG: Kyoto Encyclopedia of Gene and Genome
Nr: NCBI non-redundant
KOG: EuKaryotic Clusters of Orthologous Groups
GO: Gene Ontology
qRT-PCR: Quantitative real-time polymerase chain reaction
SD: Standard deviations
UV: Unit variance scaling
